# Unilateral hemilaminectomy vs. laminoplasty for the resection of spinal schwannomas: an analysis of 100 patients

**DOI:** 10.3389/fneur.2024.1383980

**Published:** 2024-05-28

**Authors:** Xiaofeng Chen, Dianhui Han, Tie Mao, Huilong Xu, Hua Guo, Haitao Ge, Xiangyi Meng, Lei Teng, Liankun Wang, Qingchun Mu, Jiabin Wang

**Affiliations:** ^1^Department of Neurosurgery, The First Affiliated Hospital of Harbin Medical University, Harbin, China; ^2^Department of Neurosurgery, Heilongjiang Provincial Hospital, Harbin, Heilongjiang, China; ^3^Department of Neurosurgery, The First Hospital of Sui Hua City, Suihua, Heilongjiang, China; ^4^Department of Neurology, Heilongjiang Province Hospital, Harbin, China; ^5^Department of Neurosurgery, Gaozhou People's Hospital, Guangdong Medical University, Guangdong, China

**Keywords:** unilateral hemilaminectomy, spinal schwannoma, laminoplasty, spine, surgery

## Abstract

**Objective:**

Spinal schwannomas are the most common intradural extramedullary tumors, and their complete removal is recommended to avoid tumor recurrence. Although laminoplasty provides a sufficient window for tumor resection, this approach may increase tissue trauma and cause postoperative instability compared with unilateral hemilaminectomy. This study aimed to compare the efficacy and clinical outcomes of the two approaches.

**Materials and methods:**

We included 100 consecutive patients who underwent unilateral hemilaminectomy or laminoplasty for resection of spinal schwannomas between January 2015 and February 2023. The patients' baseline characteristics, including sex, age, tumor location, percentage of tumor occupying the intradural space, operative time, postoperative length of hospital stay, intraoperative bleeding volume, visual analog scale score, and neurologic results, were retrospectively analyzed.

**Results:**

Hemilaminectomy patients who underwent unilateral hemilaminectomy had smaller intraoperative bleeding (*p* = 0.020) volume, shorter operative time (*p* = 0.012), and shorter postoperative length of hospital stay (*p* = 0.044). The mean VAS scores at the last follow-up were similar between the two groups (*p* = 0.658). Although the postoperative McCormick and Karnofsky Performance scores were not significantly different between the laminoplasty and unilateral hemilaminectomy groups (*p* = 0.687 and *p* = 0.649, respectively), there was a statistically significant improvement based on postoperative neurological results compared to preoperative neurological results for both groups. The incidence of postoperative complications was 5% and 11.7% in the unilateral hemilaminectomy and laminoplasty groups, respectively (*p* = 0.308).

**Conclusions:**

For spinal schwannoma resection, unilateral hemilaminectomy has more advantages than laminoplasty, including a shorter postoperative hospital stay, faster procedure, and less intraoperative blood loss while achieving the same desired result.

## Introduction

Spinal schwannomas are slow-growing benign WHO grade I nerve sheath neoplasm that arises from Schwann cells. Total resection of these tumors are the main goal of surgical treatment (1, 2). Although conventional surgical approaches such as laminoplasty provide an adequate window for tumor resection and restoration of the anatomical structure, they are associated with significant tissue trauma, postoperative deformities, pain, and spinal instability (3, 4).

Minimally invasive procedures, such as unilateral hemilaminectomy, minimize bony defects, reduce tissue trauma, and decrease the incidence of spinal instability (5, 6). However, it is unclear whether unilateral hemilaminectomy is safer and more effective than laminoplasty for the excision of intradural extramedullary schwannomas, and there have been no comparative studies on laminoplasty and hemilaminectomy approaches.

To the best of our knowledge, this is the first retrospective study to compare the clinical efficacy and outcomes of hemilaminectomy and laminoplasty for spinal schwannoma resection in 100 consecutive patients.

## Materials and methods

We retrospectively reviewed the clinical data of 100 consecutive patients who were performed either unilateral hemilaminectomy or laminoplasty for spinal schwannoma resection between January 2015 and February 2023 at the First Affiliated Hospital of Harbin Medical University. The approach used for each patient was selected according to the surgeon's preference. The inclusion criteria were patients (1) with tumors occupying < 3 motion segments of the spine, (2) operated on by the same surgeon, (3) operated on for >6 months, (4) with intact clinical data, (5) with tumor laterality, and (6) with intradural tumors. Exclusion criteria were as follows: patients with incomplete clinical data, those who were not followed up, those who were followed up for < 6 months, those with recurrent tumors, those with tumors occupying ≥3 motion segments of the spine, those with multiple tumors, and with dumbbell-shaped tumors developing in the neural foramen and outside the canal. For each case, clinicopathological data were carefully extracted from the hospital database, including age at the time of surgery, sex, vertebral level location (cervical, thoracic, and lumbar), percentage of tumors occupying the intradural space, surgical approach, neurofunctional status according to McCormick grading, total operative time, postoperative length of hospital stay, intraoperative bleeding volume, visual analog scale (VAS) and Karnofsky performance score (KPS). The percentage of tumors occupying the intradural space was calculated on magnetic using image analysis software (Image J; Wayne Rasband, National Institutes of Health) as follows: (maximum tumor area in cm^2^)/(intradural space area in the same section in cm^2^) × 100 (%). The pathologic diagnosis of intradural schwannoma following surgery was confirmed by three qualified pathologists.

### Operative technique

All patients underwent a posterior approach in the prone position. A small midline skin incision was made in accordance with the radiographic marker positioned on the spinous process where the lesion was located. In the unilateral hemilaminectomy group, the paravertebral muscles were retained to expose the laminae on one side. The supraspinal and interspinal ligaments and contralateral muscles were left undisturbed. Hemilaminectomy was performed with a combination of high-speed pneumatic drill round burrs and Kerrison rongeurs to resect the soft tissue and ligamentum flavum and create an adequate surgical corridor. For laminoplasty, paravertebral muscle dissection was bilateral, a high-speed pneumatic drill was used to resect the laminae, the supraspinal and interspinous ligaments were dissected, and the spinous process ligament complex was completely removed. Subsequently, the dura was opened and micro-neurosurgical techniques were used to resect the spinal schwannomas. The tumors were completely resected in all patients, and the affected nerve roots were cut.

Watertight spinal dural closure was performed running locked 6-0 Prolene (Ethicon Inc.). In laminoplasty, the incised laminae and spinous processes are installed and fixed using screws and connectors, and the supraspinal ligament is fixed with silk thread *in situ* sutures.

### Statistical analysis

SAS9.4 software version (SAS Inc., Cary, NCSU, USA) was used for the statistical analyses. The incidences of postoperative complications were compared and evaluated using Fisher's exact test. Sex and tumor location were compared and evaluated between the two approaches using the chi-square test. The VAS score, age, KPS score, McCormick score, duration of surgery, postoperative length of hospital stay, and amount of intraoperative bleeding were compared and analyzed using the Kruskal–Wallis test. Results were considered statistically significant for *p* values < 0.05.

## Result

### Patient demographic data

**Figure 1:**
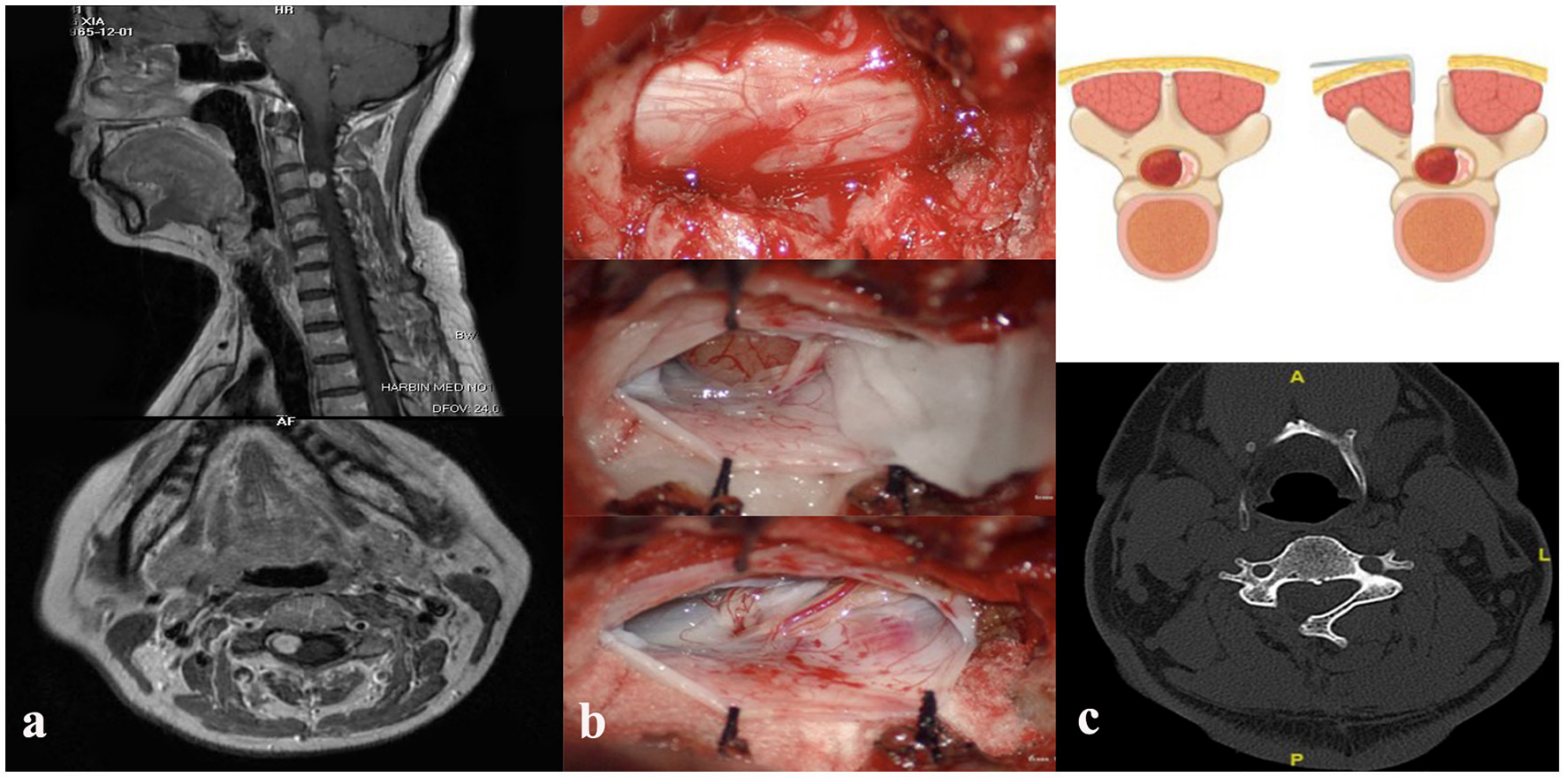
**(a)** Preoperative cervical magnetic resonance imaging T1-weighted sagittal (up) and axial (down) image with contrast showing heterogeneously enhancing intradural extramedullary lesion at the level of the C3 vertebrae. **(b)** Intraoperative unilateral hemilaminectomy with resection of the laminae (up), exposure of the tumor (middle), and removal of the lesion (down). **(c)** Demonstration of unilateral hemilaminectomy (up), postoperative computed tomography scan study (down).

**Figure 2:**
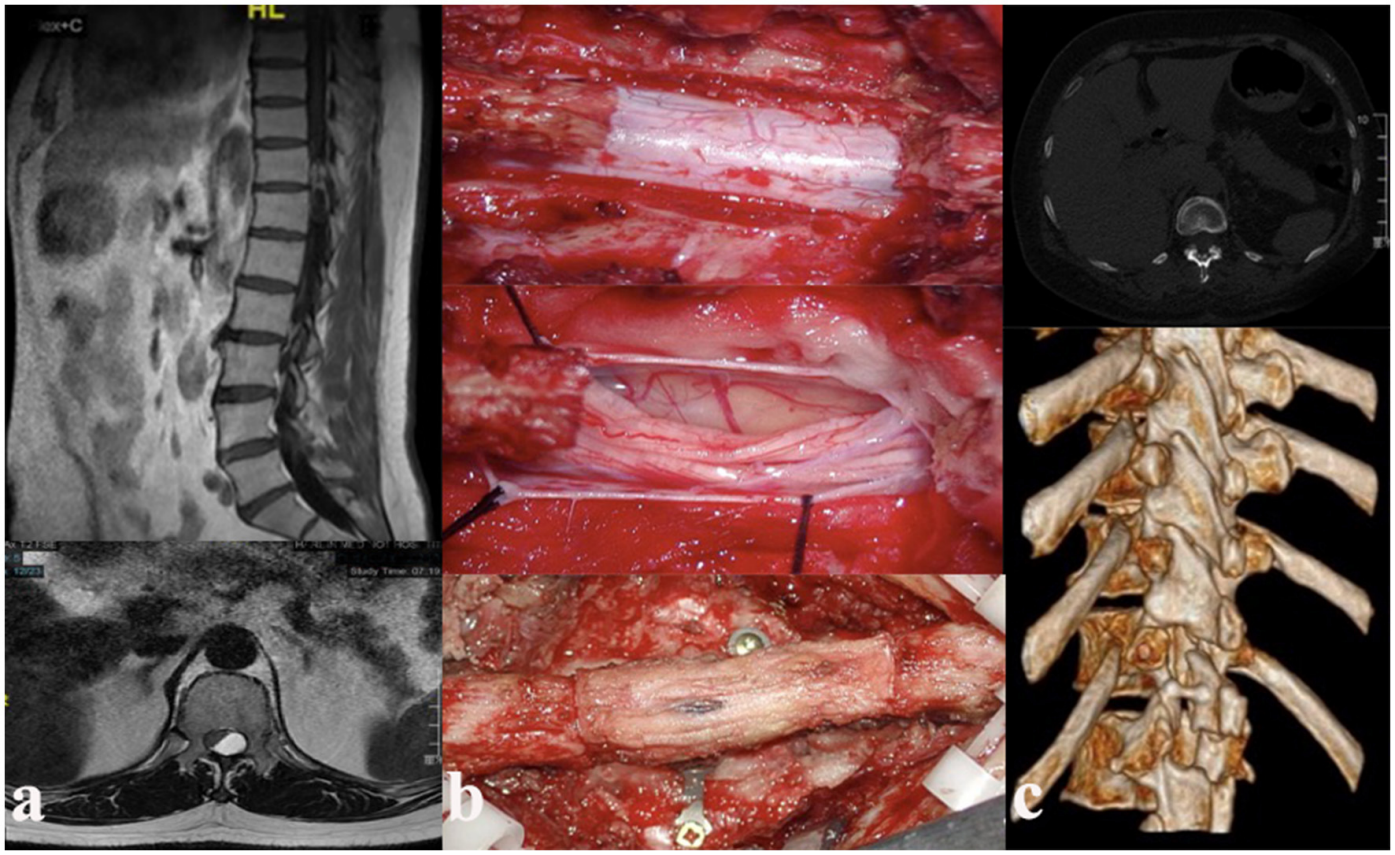
**(a)** Preoperative thoracic magnetic resonance imaging T1-weighted sagittal (up) and axial (down) image with contrast showing heterogeneously enhancing intradural extramedullary lesion at the level of the T11–12 vertebrae. **(b)** Intraoperative laminoplasty with resection of the laminae (up), exposure and resection of tumor (middle), and reduction of the resected lamina (down). **(c)** Postoperative computed tomography (CT) scan study.

The clinical data of the 100 patients were analyzed and discussed. Of these patients, 56 (56%) were women and the remaining 44 (44%) were men, with an average age of 51.2 ± 13.1 years (age range of 12–81 years). The main presenting symptoms were radiculopathy in 61 patients (61%), back pain in 14 (14%), motor deficits in 26 (26%), sensory changes in 41 (41%), and bladder/bowel dysfunction in 11 (11%). In the current study, 40 patients underwent unilateral hemilaminectomy (Figure 1), and 60 patients underwent laminoplasty (Figure 2). Age, sex, extent of tumor involvement, preoperative VAS score, preoperative McCormick score, and preoperative KPS score were not significantly different between the two groups (*p* > 0.05). In the laminoplasty group, the tumors were located in the thoracic (18.3%), cervical (38.3%), and lumbar (43.3%) spinal regions. Patients who underwent unilateral hemilaminectomy more commonly had tumors in the cervical region; 17 (42.5%) patients who underwent unilateral hemilaminectomy also had tumors in this region (Table 1). The percentage of tumors occupying the intradural space was 77.1% in the unilateral hemilaminectomy group and 82.3% in the laminoplasty group, respectively (*p* = 0.080) (Table 1).

**Table 1:** Comparison of the demographic and clinical data of patients treated for spinal schwannoma with unilateral hemilaminectomy and laminoplasty.

	**Hemilaminectomy (*n =* 40)**	**Laminoplasty (*n =* 60)**	***p* value**
Age (years), mean ± SD	53 (46–59)	52 (40.5–59.5)	0.563
**Sex**			
Male	15 (37.5)	29 (48.33)	0.285
Female	25 (62.5)	31 (51.67)	
**Site of surgery**			
Cervical	17 (42.5)	23 (38.33)	0.15
Thoracic	10 (25)	16 (18.3)	
Lumbar	13 (32.5)	21 (43.3)	
**Preoperative McCormick**			
I	25 (62.5)	41 (68.33)	0.637
II	11 (27.5)	11 (18.33)	
III	2 (5)	8 (13.33)	
IV	2 (5)	0 (0)	
Preoperative KPS score	80 (70–80)	80 (70–80)	0.825
Preoperative VAS score	6 (3.5–7)	7 (4–7)	0.236
Occupying (%)	77.1 (67.6–80.65	82.3 (68.4–84.1)	0.08
Cranio-caudal tumor extension			0.328
1 level	23(57.5%)	32(53.3%)	
2 levels	17 (42.5%)	28 (46.7%)	

### Surgical outcomes

In the current study, the operative time was significantly different between the two approaches (hemilaminectomy: 210 (172.5–247) min, laminoplasty: 232.5 (205–270) min; *p* = 0.012). Similarly, there were significant differences in intraoperative bleeding volume (hemilaminectomy: 50 (30–80) ml, laminoplasty: 70 (60–100); *p* = 0.020) and postoperative length of hospital stay (hemilaminectomy: 7 (6–9) days, laminoplasty: 9 (7–10) days; *p* = 0.044). The incidences of postoperative complications were 5% in the unilateral hemilaminectomy group and 11.67% in the laminoplasty group, respectively (*p* = 0.308) (Table 2).

**Table 2:** Surgical outcomes according to the surgical intervention.

	**Hemilaminectomy (*n =* 40)**	**Laminoplasty (*n =* 60)**	***p* value**
**Postoperative complications**			
No	38 (95)	53 (88.33)	0.308
Yes	2 (5)	7 (11.67)	
**Follow-up McCormick score**			
I	35 (87.5)	54 (90)	0.687
II	3 (7.5)	4 (6.67)	
III	2 (5)	2 (3.33)	
Follow-up KPS	90 (90–90)	90 (90–100)	0.649
Follow-up VAS	1 (0–1)	1 (0–1)	0.658
Operative time (min)	210 (172.5–247)	232.5 (205–270)	0.012
Hospitalization length (d)	7 (6–9)	9 (7–10)	0.044
Blood loss (ml)	50 (30–80)	70 (60–100)	0.02

### Follow-up and functional outcome

The average follow-up time was 49.6 ± 30.0 months (range, 6–96 months) in the laminoplasty group and 48.0 ± 28.1 months (range, 6–96 months) in the unilateral hemilaminectomy group (*p* > 0.05).

The VAS scores were evaluated in both groups, and patients reported significantly less pain at the last follow-up than at admission (*p* < 0.05) (Table 3). Remarkably, all patients experienced pain relief; however, there was no statistically significant difference in the postoperative VAS between the laminoplasty patients and unilateral hemilaminectomy patients at the last follow-up (*p* = 0.658) (Table 2).

**Table 3:** Comparison of VAS and KPS scores according to the surgical intervention type.

	**Preoperative**	**Follow-up**	***p* value**
**Hemilaminectomy (*****n** =* **40)**			
VAS	6 (3.5–7)	1 (0–1)	0.005
KPS	80 (70–80)	90 (90–90)	0.03
**Laminoplasty (*****n** =* **60)**			
VAS	7 (4–7)	1 (0–1)	0.004
KPS	80 (70–80)	90 (90–100)	0.04

The KPS and McCormick scores reflect the patients' functional outcomes. In the unilateral hemilaminectomy group, the overall median KPS improved from an average of 80 (70–80) to 90 (90–100) (*p* = 0.03), whereas in the laminoplasty group, it improved from an average of 80 (70–80) to 90 (90–90) (*p* = 0.040) (Table 3). In terms of neurological recovery based on the McCormick grade, 11 (27.5%) patients in the unilateral hemilaminectomy group and 16 (26.7%) patients in the laminoplasty group showed improvement, while 29 (72.5%) patients in the unilateral hemilaminectomy group and 43 (71.7%) patients in the laminoplasty group showed no improvement (Table 4). In the laminoplasty group, 1 patient with McCormick grade II deteriorated to McCormick grade III. Although there was no statistically significant difference at the last follow up in the postoperative McCormick score between the laminoplasty group and unilateral hemilaminectomy group (*p* = 0.687), there was a statistically significant difference in the postoperative McCormick score compared to the preoperative McCormick score in both groups (hemilaminectomy, *p* = 0.0005; laminoplasty, *p* = 0.0002) (Table 5), indicating that patients in both groups experienced significant improvement in neurological function. None of the patients developed iatrogenic kyphosis requiring fusion or instrumentation.

**Table 4:** Comparison of postoperative neurological outcomes between patients treated with hemilaminectomy or laminoplasty at the last follow-up.

	**Hemilaminectomy**	**Laminoplasty**	***p* value**

	**(*****n** =* **40)**	**(*****n** =* **60)**	
Neurologically improved *n* (%)	11 (27.5)	16 (26.7)	0.824
Neurologically the same *n* (%)	29 (72.5)	43 (71.)	
Aggravated_ neurologically *n* (%)	0 (0)	1 (1.6)	

**Table 5:** Comparison of neurological outcomes according to surgical intervention type.

	**Preoperative McCormick (*n*, %)**	**Follow-up McCormick (*n*, %)**	***p* value**
**Hemilaminectomy (*****n** =* **40)**			
I	25 (62.5)	35 (87.5)	0.0005
II	11 (27.5)	3 (7.5)	
III	2 (5)	2 (5)	
IV	2 (5)	0 (0)	
**Laminoplasty (*****n** =* **60)**			
I	41 (68.33)	54 (90)	0.0002
II	11 (18.33)	4 (6.67)	
III	8 (13.33)	2 (3.33)	
IV	0 (0)	0 (0)	

## Discussion

Spinal schwannomas are slow-growing benign WHO grade I nerve sheath neoplasm arising from Schwann cells, accounting for 55% of all intraspinal tumors (7). The age of patients at the onset of spinal schwannoma symptoms was 60–70 years (8). In our study, the average age of our patients was consistent with that reported in previous literature. Total tumor excision is the gold standard treatment because it is associated with minimal morbidity and functional improvement (9). Several approaches have been accepted for the resection of spinal tumors, including laminectomy or laminoplasty. These approaches require bilateral dissection of the paraspinal muscles from the lamina (10).

Resection of the lamina and the interspinous ligaments may result in postoperative back pain and increase the risk of late-stage spinal instability or kyphosis. The incidence of post-laminectomy spinal deformities ranges from 33% to 100% (11, 12). Although laminoplasty, as an improved technique, may restore the spinal integrity of the posterior elements, both animal and clinical studies have reported a lower incidence of kyphotic deformities after laminoplasty (3).

As the main goal is complete resection, minimally invasive techniques are advantageous because they avoid iatrogenic trauma, prevent possible instability, and reduce the incidence of postoperative complications while achieving the same desired result. The unilateral hemilaminectomy has become the preferred surgical technique for the removal of intraspinal lesions (13, 14). Some authors have performed a unilateral hemilaminectomy approach for tumor resection and found the advantages of less intraoperative bleeding, fewer postoperative complications, and a shorter length of hospital stay (15, 16). In contrast, Iacoangeli et al. showed that the exposure generated by unilateral hemilaminectomy was limited, which may prolong the operative time and increase the amount of intraoperative blood loss (17). In the current study, 40% patients underwent tumor excision using unilateral hemilaminectomy. Consistent with previous studies, the present study revealed that unilateral hemilaminectomy was associated with significantly less blood loss, shorter operative time, and significantly shorter postoperative hospital stay. This may be owing to the minimal invasiveness of unilateral hemilaminectomy or its association with minimal iatrogenic trauma, including the preservation of the contralateral zygapophyseal joints, contralateral paraspinal musculature, supraspinous, and interspinous ligaments with the integrity of the “tension band” (18).

In unilateral hemilaminectomy, the narrow surgical corridor between the spinous process and the facet joint may increase the risks of inadequate closure of the dura mater and nerve injury, thereby leading to severe postoperative pain, postoperative infection, cerebrospinal fluid leak, and neurological dysfunction (19). Therefore, we compared the postoperative KPS and McCormick scores of the unilateral hemilaminectomy group with those of the laminoplasty group, and the findings revealed that there were no statistically significant in neuro-functional recovery. Moreover, in the current study, the incidence of postoperative cerebrospinal fluid leakage and infection was analyzed. The incidence of postoperative complications was 5% and 11.7% in the unilateral hemilaminectomy and laminoplasty groups, respectively. Our study revealed that compared with laminoplasty, unilateral hemilaminectomy was not associated with an increased risk of postoperative complications, which is consistent with the results of previous studies.

Previous studies have shown that compared with laminectomy, unilateral hemilaminectomy not only reduces the probability of postoperative pain but also relieves pre-existing pain (6). However, in the current study, there was no statistically significant difference in the postoperative VAS scores between the laminoplasty and unilateral hemilaminectomy groups at the last follow-up, which might have allowed anatomical reconstruction of the spinal posterior element and a longer follow-up.

Although the narrow surgical corridor was a disadvantage, the exposed operative field was adequate for microsurgery; in particular, undercutting of the spinous process base and oblique tilting of the operating table allowed safe reversal of spinal schwannomas.

This study has some limitations, such as its retrospective nature, and the choice of approach for each case was based mainly on the operator's experience. Furthermore, the time for postoperative VAS measurement and postoperative neurological testing were not uniform for all patients. Therefore, further prospective randomized studies with large populations that include both unilateral hemilaminectomy and laminoplasty techniques to verify the results of the present study are needed.

However, compared to patients who underwent laminoplasty, those who underwent unilateral hemilaminectomy had a shorter operative time, less blood loss, and faster recovery. More importantly, the differences in complication rates and long-term functional outcomes between the two techniques were not statistically significant.

## Conclusion

Unilateral hemilaminectomy is advantageous for the resection of spinal schwannomas. Our findings show that this procedure allows safe, effective, and complete removal of intradural tumors with satisfactory outcomes and many benefits, such as a shorter hospital stay, shorter operative time, and less blood loss.

## Data availability statement

The original contributions presented in the study are included in the article/supplementary material, further inquiries can be directed to the corresponding authors.

## Ethics statement

The studies involving humans were approved by the First Affiliated Hospital of Harbin Medical University. The studies were conducted in accordance with the local legislation and institutional requirements. The participants provided their written informed consent to participate in this study. Written informed consent was obtained from the individual(s) for the publication of any potentially identifiable images or data included in this article.

## Author contributions

XC: Methodology, Writing – review & editing. DH: Formal analysis, Methodology, Writing – original draft. TM: Formal analysis, Investigation, Methodology, Writing – original draft. HX: Data curation, Formal analysis, Writing – review & editing. HGu: Formal analysis, Project administration, Resources, Writing – original draft, Writing – review & editing. HGe: Formal analysis, Writing – review & editing. XM: Formal analysis, Investigation, Writing – original draft, Writing – review & editing. LT: Methodology, Project administration, Writing – original draft. LW: Writing – review & editing, Funding acquisition. QM: Funding acquisition, Writing – review & editing. JW: Formal analysis, Funding acquisition, Writing – original draft, Writing – review & editing.
